# Arabinogalactan-proteins of the eusporangiate fern *Psilotum nudum* show atypical structural features compared to other ferns

**DOI:** 10.1016/j.tcsw.2025.100157

**Published:** 2025-10-16

**Authors:** Kim-Kristine Mueller, Lukas Pfeifer, Urska Repnik, Marc Bramkamp, Birgit Classen

**Affiliations:** a Pharmaceutical Institute, Department of Pharmaceutical Biology, Kiel University, 24118 Kiel, Germany; b Central Microscopy Facility, Kiel University, 24118 Kiel, Germany; c Institute for General Microbiology, Kiel University, 24118 Kiel, Germany

**Keywords:** *Arabidopsis thaliana*, Arabinogalactan-protein, Cell wall, JIM13, *Psilotum nudum*, Pyranosidic arabinose, Whisk fern

## Abstract

During the colonization of land, abiotic and biotic challenges induced strong adaptations in plants, including changes of their cell walls. Up to now, cell walls of spore-bearing plants have been only sparsely investigated. As ferns are the sister group to seed plants, studying their cell walls is key to understanding the evolutionary development of the cell wall in the tracheophyte lineage. Beside polysaccharides, especially arabinogalactan-proteins (AGPs) are important cell wall components involved in structural but also signalling functions. We chose a member of the eusporangiate ferns, *Psilotum nudum*, to characterize and immunolocalize AGPs. Biochemical analyses identified special structural features of *P. nudum* AGPs in comparison to *A. thaliana* AGPs, including lower amounts of 1,6-linkage in the galactan core. With regard to the arabinose (Ara) residues, *A. thaliana* AGP was dominated by terminal furanosidic Ara, followed by 1,5-Ara, whereas in *P. nudum* AGPs, 1,3-Ara, terminal furanosidic Ara and terminal pyranosidic Ara occurred in comparable amounts. Terminal pyranosidic rhamnose was only found in the fern AGP. Immunofluorescence analysis using JIM13 antibody detected AGPs in all tissues of the *P. nudum* stem with the exception of the mature protoxylem. Localization was observed along the plasma membrane as well as tonoplast.

## 1 Introduction

Ferns are the second most species-rich clade of vascular plants after angiosperms and colonize a wide variety of habitats and ecosystems (Marchant et al., 2022; Ali et al., 2025). The common ancestors of ferns and seed plants evolved around 360 million years ago. Based on the structure of the sporangia walls, ferns are subdivided today into eusporangiate and leptosporangiate species. The sporangium wall of eusporangiate ferns consists of two or more layers, while that of leptosporangiates has only one layer (Pryer et al., 2004; Popper, 2006).

**Fig. 1:**
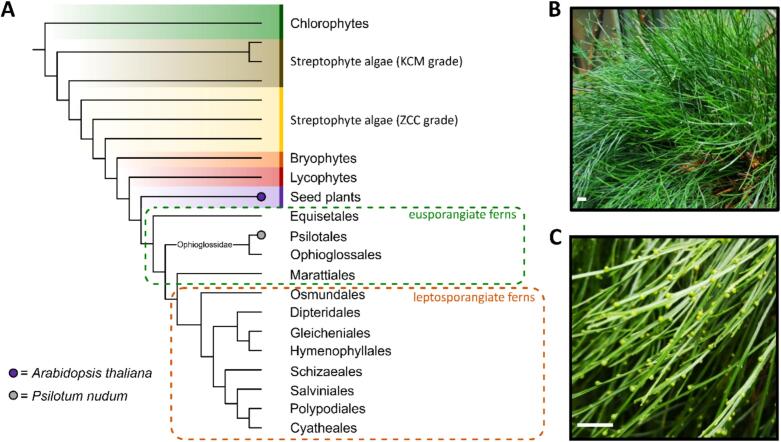
Phylogeny and habitus of *Psilotum nudum*. **A**: Cladogram of major lineages within Viridiplantae, including eusporangiate and leptosporangiate fern classification (according to Pryer et al., 2004 and Ali et al., 2025). The position of the investigated species is indicated by the dots. **B**: Individuum of *P. nudum*. **C**: Shoots with synangia. Scale bars: 1.0 cm.

The main difference between ferns and seed plants is the dependency of the gametophyte on the sporophyte. Whereas in ferns both generations are almost completely independent of each other, the gametophyte of seed plants is completely dependent on the sporophyte. Phylogenetically, ferns are the sister group to seed plants (see Fig. 1A for the phylogenetic tree) and are therefore important for studying the cell wall evolution within tracheophytes (Delaux et al., 2019; Fang et al., 2022; Ali et al., 2025).

*Psilotum nudum* (L.) Beauv. is an eusporangiate fern of the genus *Psilotum* and a member of the family Psilotaceae (Fig. 1B). This family contains only two genera: *Psilotum* and *Tmesipteris* (Šamec et al., 2019). *P. nudum* is perennial and native to tropical, subtropical and rocky landscapes or occurs epiphytically (Brownsey and Lovis, 1987; Chernova et al., 2020). The phenotype of *P. nudum* differs from most other ferns in that *P. nudum* lacks true roots and vascularized leaves. Instead, this fern morphologically consists of aerial shoots and an underground rhizome part. Individual shoots resemble a stem and are highly forked, from which the common name whisk fern originates, which is typical for this genus (Valavan et al., 2016; Chernova et al., 2020). The branched shoots have enations, which are tiny avascular scale-like appendages. Located above these enations are clusters of sporangia, termed synangia (Fig. 1C). These arise from the fusion of three neighboring sporangia and contain the spores (Šamec et al., 2019). The rhizome bears rhizoids and can be symbiotic with mycorrhizal fungi (Šamec et al., 2019). The simplified body plan of *P. nudum*, resembling some of the earliest fossil lineages of vascular plants (e.g. *Rhynia*, Pryer et al., 2004), was long thought to indicate a close relationship to the latter and led to the denomination of *P. nudum* as a “living fossil” (Chernova et al., 2020). Today, it seems to be accepted that the unique morphology is secondary and based on a reduction of characters (Ruhfel et al., 2014).

Phylogenetically, extant individuals belong to a clade separated from all other ferns by the earliest split within the lineage (Ali et al., 2025). Consequently, *P. nudum* is of special interest for evolutionary research on tracheophyte cell walls.

The plant cell wall is a highly dynamic, complex structure, which is ubiquitous in the plant kingdom. It consists mainly of polysaccharides, cell wall glycoproteins and sometimes polyphenols, mainly lignin. The three main classes of plant cell wall polysaccharides are cellulose, hemicelluloses and pectins (Popper, 2008; Mohnen, 2008; Scheller and Ulvskov, 2010; Barnes et al., 2021). In addition to the high proportion of polysaccharides, there are also cell wall glycoproteins, mostly hydroxyproline-rich-glycoproteins (HRGPs). The HRGPs with the highest degree of glycosylation (up to 90%) are the arabinogalactan-proteins (AGPs; Ellis et al., 2010). The carbohydrate part consists mainly of Ara and Gal and contains minor amounts of other monosaccharides in its side chains. The core structure is a (1 → 3)-β-d-galactan chain that is linked to (1 → 6)-β-d-galactan side chains (Ma et al., 2018) and these are frequently decorated with arabinose residues. The (1 → 3)-β-d-galactan chain is responsible for one of the special features of AGPs: the precipitation with the β-glucosyl Yariv reagent (βGlcY), which can be used to isolate AGPs (Yariv et al., 1962; Paulsen et al., 2014; Ma et al., 2018). The polysaccharide part is covalently linked to the protein part via the amino acid hydroxyproline (Hyp; Ellis et al., 2010). AGPs are involved in a variety of functions in seed plants, including cell proliferation and division, growth, pattern formation, xylem differentiation, programmed cell death or the regulation of salt stress (Seifert and Roberts, 2007; Lamport et al., 2014; Ma et al., 2018; Pfeifer et al., 2020).

In recent years, AGPs from several bryophytes and ferns have been isolated and characterized by immunocytochemical and analytical studies (Carafa et al., 2005; Fu et al., 2007; Eeckhout et al., 2014; Leroux et al., 2015; Bartels et al., 2017; Bartels and Classen, 2017; Happ and Classen, 2019; Chernova et al., 2020; Mueller et al., 2023). However, until now, AGPs of only one eusporangiate fern (*Equisetum*) have been isolated and characterized (Bartels and Classen, 2017); therefore, this study expands knowledge on AGPs in basal ferns and furthers understanding of the evolution of AGPs in the tracheophytes.

## 2 Materials and methods

### 2.1 Plant material, growth conditions and sampling

Sporophytes of *Psilotum nudum* (L.) Beauv. (*Psilotum*, *P. nudum*) and *Arabidopsis thaliana* (L.) Heynh. (*Arabidopsis*, *A. thaliana*) were grown in the greenhouse (*P. nudum*, harvest time in August 2020) and in the garden (*A. thaliana*, harvest time in May 2023) of the Pharmaceutical Institute of Kiel University. For sampling, the areal shoots of *P. nudum*, and the above ground parts of *A. thaliana* were collected, washed with water and freeze-dried.

### 2.2 Isolation of arabinogalactan-proteins

The preparation of the aqueous extract (AE) for *P. nudum* in a ratio of 1:20 (*w/V*) and for *A. thaliana* in a ratio of 1:10 (*w/V*) as well as isolation of AGPs from both plants has been performed according to Mueller et al. (2023).

### 2.3 Determination of the hydroxyproline (Hyp) and protein content of the AGPs

The Hyp content was quantified photometrically at 558 nm by a linear regression analysis according to Stegemann and Stalder (1967). The protein content was calculated via the nitrogen content determined by elemental analysis (multiplication factor 6.25; Kjeldahl, 1883). Nitrogen analysis was conducted with the vario MICRO cube elemental analyzer (Elementar Analysensysteme GmbH, Langenselbold, Germany) in the Chemistry Department of Kiel University**,** Kiel, Germany.

### 2.4 Analysis of monosaccharides

Neutral monosaccharide composition was determined by the method of Blakeney et al. (1983) with slight modifications, described in Mueller et al. (2023). The quantification was performed by gas chromatography (GC) with flame ionization detection (FID) and mass spectrometry detection (MSD): GC + FID: 7890B; Agilent Technologies, USA; MS: 5977B MSD; Agilent Technologies, USA; column: Optima-225; Macherey-Nagel, Germany; 25 m, 250 μm, 0.25 μm; split ratio 30:1; helium flow rate: 1 ml min^−1^. A temperature gradient was used to achieve peak separation (initial temperature 200 °C, subsequent holding time of 3 min; final temperature 243 °C with a gradient of 2 °C min^−1^).

The content of uronic acids was determined photometrically at 525 nm by a modified method of Blumenkrantz and Asboe-Hansen, 1973 (see Mueller et al., 2023).

### 2.5 Reduction of uronic acids and partial degradation of AGPs

To obtain the AGP_UR_ fraction, the uronic acid residues, which are not detectable by the method described above (“Analysis of monosaccharides”), were reduced to the corresponding neutral monosaccharides following a modified method of Taylor and Conrad (1972). A part of the AGP_UR_ fraction was partially degraded by oxalic acid hydrolysis (Gleeson and Clarke, 1979) to gather further structural information (AGP_UROx_ fraction). For details follow the description in Mueller et al. (2023).

### 2.6 Structural characterization of arabinogalactan moieties of AGPs

The AGP, the AGP_UR_ and the AGP_UROx_ fractions of *P. nudum* and *A. thaliana* were used for structure elucidation. Therefore, a modified method of Harris et al. (1984), described in Mueller et al. (2023) was applied to obtain permethylated alditol acetates (PMAAs). The quantification of those PMAAs were performed by GLC-mass spectroscopy (see section”Analysis of monosaccharides”; column: Optima-1701, 25 m, 250 μm, 0.25 μm; helium flow rate: 1 ml min^−1^; initial temperature: 170 °C; hold time 2 min; rate 1 °C min^−1^ until 210 °C was reached; rate: 30 °C min^−1^ until 250 °C was reached; final hold time 10 min).

### 2.7 Indirect enzyme-linked immunosorbent assay (ELISA)

The samples of the AGP fractions were dissolved in ddH_2_O at concentrations of 12.5, 25 and 50 μg ml^−1^. The test was performed according to Pfeifer et al. (2023). The primary antibodies JIM13, LM2 and LM6 were purchased from Kerafast (Inc., Boston, MA, USA); for KM1 see Classen et al., 2004. All of them were used at a 1:20 (*V/V*) dilution. The secondary antibody anti-rat-IgG was used for the Kerafast primary antibodies, an anti-mouse-IgG antibody for the KM1 antibody. Both secondary antibodies were conjugated with alkaline phosphatase (produced in goat, Sigma-Aldrich Chemie GmbH, Taufkirchen, Germany) and dissolved at a 1:500 (*V/V*) dilution. The assay was performed in triplicate.

### 2.8 Histochemistry staining for brightfield microscopy

Cross sections of the *P. nudum* stem (1.2–1.5 mm in diameter) were prepared using a home-made microtome device and stained with Sudan III, toluidine blue or phloroglucinol / HCl. Other sections were incubated overnight at 4 °C in a solution of βGlcY, or α-Gal-Yariv (αGalY) as negative control (both 1 mg ml^−1^ in 0.15 mol l^1^ sodium chloride). The sections were washed with 0.15 moll^−1^ sodium chloride solution until the red colour disappeared. Unstained and stained sections were investigated by light microscopy (Primostar 3, Carl Zeiss Microscopy Deutschland GmbH, Oberkochen, Germany) and the images taken by a digital camera (Axiocam 208 colour, Carl Zeiss Microscopy Deutschland GmbH).

### 2.9 JIM13 labelling for fluorescence microscopy

Stems with 1.2–1.5 mm in diameter were cut into 3–5 mm long segments and transferred to 4% formaldehyde in 50 mmol l^−1^ phosphate buffer, pH 7.2, supplemented with 1 mmol l^1^ MgCl_2_ and 1 mmol l^−1^ CaCl_2_. The air trapped inside the tissue was released by transferring the plant segments into a 10 ml syringe and creating low vacuum by pulling a plunger while holding a finger over the syringe tip to seal it. Tissue was transferred back into the original container and incubated with the fixative for 2 d at room temperature with agitation. Thereupon the fixative was replaced with PBS and stored at 4 °C. To prepare 60–80 μm vibratome sections, stem segments were embedded into 4% agarose and cut using a Zeiss Hyrax V50 vibratome. During the immunolabelling procedure, sections were incubated in microtubes. First, sections were washed with 50 mmol l^−1^ ammonium chloride in PBS to quench any residual fixative, followed by incubation with 0.5% saponin in PBS for 20 min, and then with the blocking buffer (1% cold water fish skin gelatin in PBS) for 35 min. The JIM13 primary antibody (rat IgM; Kerafast) was diluted 1:10 with the blocking buffer and incubated with sections for 1 h in a thermoblock at 22 °C with 400 rpm agitation. After washing with PBS, sections were incubated with the secondary goat anti-rat IgM antibody conjugated to DyLight550 (Thermo Fisher Scientific), diluted 1:250 with the blocking buffer (final conc. of 2 μg ml^−1^), for 45 min. After washing with PBS, sections were mounted under coverslips using Mowiol 4–88 mounting medium. For the negative control, sections were incubated only with the secondary antibody.

Samples were analysed with an LSM900 with Airyscan microscope using a 20×/0.8 NA Plan Apochromat objective and ZEN 3.2 (blue edition) software (all Zeiss). The DyLight 550 fluorophore was visualized with 561 nm excitation and 565–600 nm detection. Chlorophyll B was visualized with 640 nm excitation and 645–700 detection. For both steps, GaAsP detector in the super-resolution Airyscan mode was used. *Z*-stacks (2-μm interval) of tiled images were processed using the Zen software. Transmitted light images were acquired using the 640 nm excitation and the ESID detector.

### 2.10 Principal component analysis (PCA)

The results of the linkage-type analyses were used for principal component analysis (PCA) of AGP samples throughout the green lineage (streptophyte algae, bryophytes, lycophytes, ferns, gymnosperms and angiosperms). The built-in functions in R Studio (version 2024.12.1 + 563), as well as the ggfortify package (Tang et al., 2016) were used for generation of scaled and centred biplots (PC1 vs. PC2).

## 3 Results

### 3.1 Water-soluble polysaccharides and arabinogalactan-proteins present in the cell walls of *P. nudum* and *A. thaliana*

**Fig. 2:**
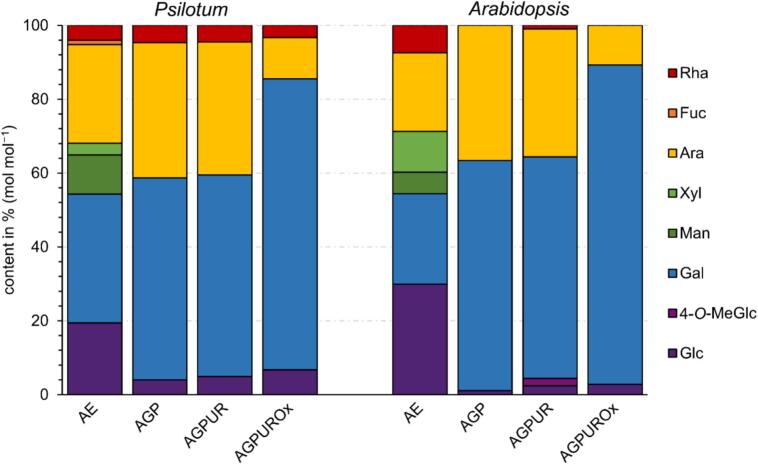
Neutral monosaccharide composition of the aqueous extracts (AE), the native AGPs, AGPs after reduction of uronic acids (AGP_UR_) and subsequent partial acid hydrolysis (AGP_UROx_) determined by gas chromatography (GC;% mol mol^−1^) from *Psilotum nudum* and *Arabidopsis thaliana*. Detailed data are shown in Tables S1 and S2. Legend abbreviates: Rha – rhamnose; Fuc – fucose; Ara –arabinose; Xyl – xylose; Man – mannose; Gal – galactose; 4-*O*-MeGal – 4-*O*-methyl glucose; Glc – glucose. Neutral monosaccharide composition of the aqueous extracts (AE), the native AGPs, AGPs after reduction of uronic acids (AGP_UR_) and subsequent partial acid hydrolysis (AGP_UROx_) determined by gas chromatography (GC;% mol mol^−1^) from *Psilotum nudum* and *Arabidopsis thaliana*. Detailed data are shown in Tables S1 and S2. Legend abbreviates: Rha – rhamnose; Fuc – fucose; Ara –arabinose; Xyl – xylose; Man – mannose; Gal – galactose; 4-*O*-MeGal – 4-*O*-methyl glucose; Glc – glucose.

To make a direct comparison between the *P. nudum* fern and a seed plant, we also investigated the model plant *A. thaliana* in this study. The freeze-dried plant material of *P. nudum* and *A. thaliana* was extracted with water to isolate a water-soluble fraction (AE), which potentially contained AGPs. The yield of the AE of *P. nudum* amounted to 2% and for *A. thaliana* to 5% of dry plant material (w w^−1^). The neutral monosaccharide compositions are shown in Fig. 2 and Table S1 and S2. In AEs of both *P. nudum* and *A. thaliana*, Glc, Ara and Gal were present in high amounts between 19.4% and 34.9%. Man, Xyl and Rha occurred in minor amounts between 3.2% and 11.1%. In both fractions, the absolute uronic acid content was similar (3.0% for *P. nudum*, 3.7% for *A. thaliana*).

As both AEs contained high amounts of Ara and Gal, precipitation with the βGlcY reagent was used to obtain water soluble AGPs. The AGP fraction of *P. nudum* accounted for 0.08%, and of *A. thaliana* for 0.16% of the dry plant material (w w^−1^). The neutral monosaccharide compositions of both AGP fractions are shown in Fig. 2 and Table S1 and S2. The Ara + Gal content of both was high, with 91.3% for *P. nudum* AGP and 98.9% for *A. thaliana* AGP. The Ara: Gal ratio was slightly different (1, 1.5 for *P. nudum* and 1, 1.7 for *A. thaliana*). Whereas *P. nudum* AGP in addition contained 4% of Glc and 4.7% of Rha, only 1.1% of Glc and traces of Rha were detected in *A. thaliana* AGP. Low amounts of Glc might have originated from the βGlcY reagent. The contents of the uronic acids were quite similar with 6.9% of the fern and 7.0% of the dicotyl AGP.

To get further insights in the nature of uronic acids, their carboxyl groups were reduced with sodium boron deuteride to gain the AGP_UR_ fraction. The neutral monosaccharide compositions of both AGP_UR_ fraction are demonstrated in Fig. 2 and Tables S1 and S2. The *P. nudum* AGP_UR_ as well as the *A. thaliana* AGP_UR_ showed a small increase of the Glc amount compared to their content in the original AGP preparation. Deuterated fragments were identified among Glc monomers by mass spectrometry analysis, confirming the presence of GlcA in both native AGPs. In addition, in the AGP_UR_ from *A. thaliana*, an unknown peak was found and identified as 4-*O*-Me-GlcA by mass spectrometry according to Pfeifer et al. (2020).

To gain more structural information with regard to the side chains organisation, a mild acid partial hydrolysis was performed. In this reaction, labile Ara residues were hydrolysed from the galactan core structure (AGP_UROx_). In both AGP_UROx_ preparations, Ara content decreased to approximately one third of the pre-hydrolysis values (Fig. 1 and Table S1 and S2).

In AGPs, the amino acid hydroxyproline (Hyp) covalently links the carbohydrate moiety with a protein part. The amount of protein was determined by elemental analysis of the nitrogen content using the multiplication factor of 6.25 (Kjeldahl, 1883). The nitrogen content was 0.86% (*w/w*) for *P. nudum* AGP, and 1.14%(*w/w*) for *A. thaliana* AGP, which translates into the protein content of 5.4% for *P. nudum*, and 7.1% for *A. thaliana*. Hyp was measured photometrically, yielding an absolute Hyp content of 0.33% (*P. nudum*) and 0.62% (*A. thaliana*). Calculating this Hyp amount in relation to the protein, the Hyp content was 6.1% of the protein moiety in *P. nudum* AGP, and 8.7% of the protein moiety in *A. thaliana* AGP.

### 3.2 Elucidation of arabinogalactan-fine structures of the AGPs

**Fig. 3:**
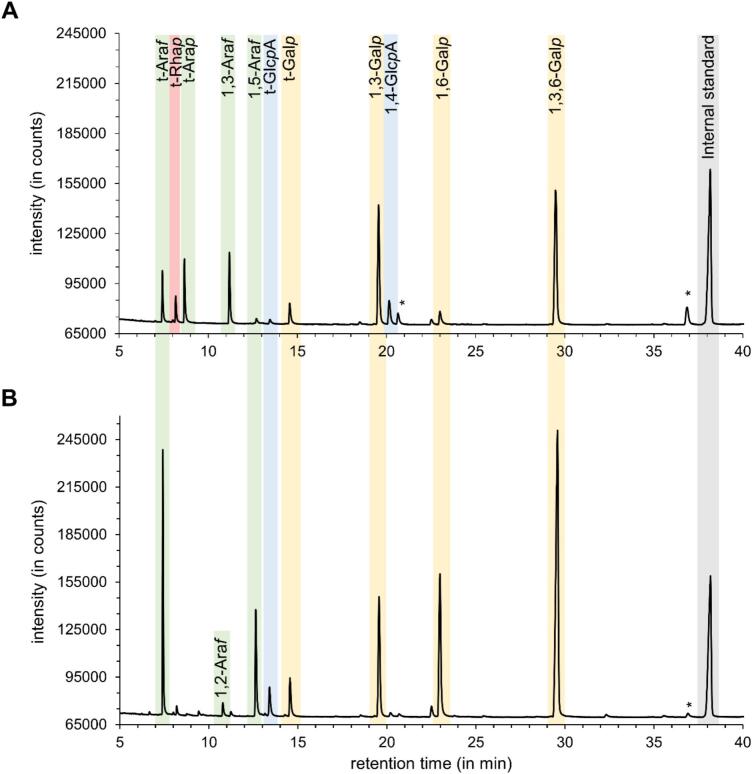
FID chromatograms of permethylated alditol acetates of uronic acid-reduced AGPs (AGPs_UR_) from *Psilotum nudum* (**A**) and *Arabidopsis thaliana* (**B**) after GLC analysis (*incompletely methylated constituents).

The structural elucidation was performed with the native AGP, the AGP_UR_ and the AGP_UROx_ by linkage type analysis (Table 1, Table 2). The FID chromatograms of permethylated alditol acetates (PMAAs) of AGPs_UR_ are shown in Fig. 3A and B.

**Table 1:** Linkage type analysis of AGP, AGP_UR_, and AGP_UROx_ of *Psilotum nudum* in% (mol mol^−1^).

Mono-saccharide	linkage type	AGP	AGP_UR_	AGP_UROx_
Gal*p*	1,3,6-	34.4	33.5	31.5
	1,6-	3.4	2.9	17.7
	1,3-	21.9	20.5	18.6
	1-	3.5	4.2	14.3
Glc*p*	1-	tr	1.1⁎	1.9⁎
	1,4-	3.4	4.9⁎	4.4⁎
Man*p*	1,6-	1.1	1.1	tr
Rha*p*	1-	3.6	3.4	3.0
Fuc*p*	1-	–	–	–
Ara*f*	1,5-	1.4	1.1	–
	1,3-	10.3	10.8	3.6
	1-	7.1	7.1	1.5
Ara*p*	1-	9.9	9.5	3.5

tr: trace value 0.4% – 1%; linkage types <0.4% not listed.

⁎ including deuterated fragments originating from reduction of GlcA.

**Table 2:** Linkage type analysis of AGP, AGP_UR_, and AGP_UROx_ of *Arabidopsis thaliana* in% (mol mol^−1^).

Mono-saccharide	linkage type	AGP	AGP_UR_	AGP_UROx_
Gal*p*	1,3,6-	37.4	39.4	28.4
	1,6-	13.5	14.7	39.2
	1,3-	10.5	10.9	12.6
	1-	3.9	3.8	9.2
Glc*p*	1-	tr	2.9⁎	3.8⁎
	1,4-	–	tr	tr
Man*p*	1,6-	2.1	1.1	tr
Rha*p*	1-	tr	tr	tr
Fuc*p*	1-	–	tr	–
Ara*f*	1,5-	10.0	9.0	1.3
	1,3-	tr	tr	–
	1,2-	1.2	1.2	tr
	1-	21.4	17.0	5.5
Ara*p*	1-	–	–	–

tr: trace value 0.4% – 1%; linkage types <0.4% not listed.

⁎ including deuterated fragments originating from reduction of GlcA

In AGPs from both *P. nudum* and *A. thaliana* (AGP without, AGP_UR_ with uronic acids), the linkage types of the galactan framework included 1,3,6-Gal*p,* 1,3-Gal*p,* 1,6-Gal*p* and terminal Gal*p*. However, there were differences in the quantities of these linkage types: in *P. nudum* AGP, the content of 1,3-Gal*p* was twice that in *A. thaliana* AGP, while the content of 1,6-Gal*p* amounted to only one fifth (Table 1, Table 2). In addition, differences between the two species were observed in the Ara linkage types. In *A. thaliana* AGP, the dominant linkage was the terminal Ara*f*, followed by 1,5-Ara*f*. In *P. nudum* AGP, terminal Ara*f* was one of three dominant linkage types along with terminal Ara*p* and 1,3-Ara*f*. The latter two linkages were not characteristic for *A. thaliana* AGP. Terminal Glc*p*A was present in both AGPs at trace values, while 1,4-Glc*p* occurred additionally in *P. nudum*. Low amounts of terminal Rha*p* were detected only in *P. nudum* AGP.

**Fig. 4:**
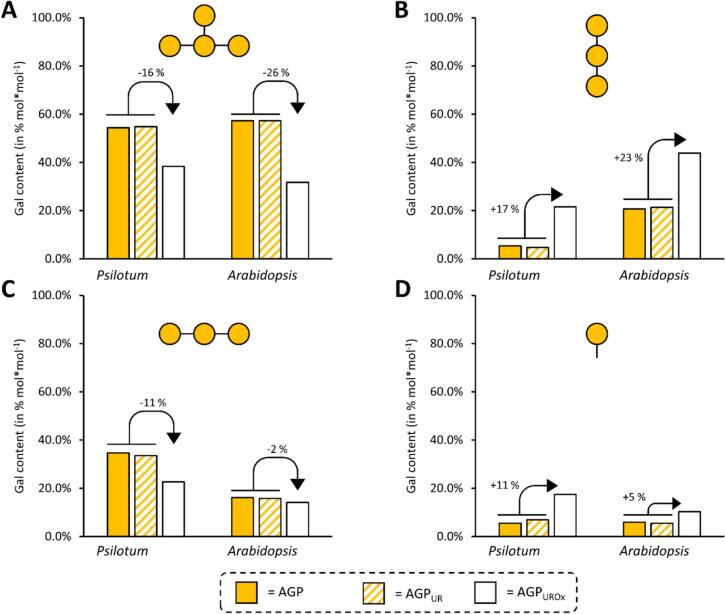
Results of the linkage-type analysis with a focus on galactose residues in AGPs of both plant species *Psilotum nudum* and *Arabidopsis thaliana*. (**A-D**) show the relative content of galactose before (AGP) and after uronic acid reduction (AGP_UR_) and with additional oxalic acid hydrolysis (AGP_UROx_). Relative decrease in 1,3,6-Gal*p* (**A**) and 1,3-Gal*p* (**C**) as well as relative increase in 1,6-Gal*p* (**B**) and t-Gal*p* (**D**) are expressed as percentages (relative to mean prior to hydrolysis).

The partially degraded AGP_UROx_ fractions of both plants revealed a decrease in all Ara linkage types and an increase in Gal*p* linkages to more than 80%. Fig. 4 shows changes in the content of Gal linkage types after reduction of uronic acids (AGP_Ur_) and partial hydrolysis (AGP_UrOx_). As expected, these remained comparable in AGP and AGP_Ur_. The decrease in 1,3,6-Gal*p* together with the strong increase in 1,6-Gal*p* after partial acid hydrolysis (Fig. 4) indicates that Ara is linked to position C3 of 1,3,6-Gal*p* in both native AGPs. The decrease in 1,3-linked Gal especially in *P. nudum* AGP_UrOx_ might be a hint that the 1,3–1inked galactan backbone is interrupted by some acid labile Ara*f* residues (“kinked” regions; Churms and Stephen, 1984).

### 3.3 Identification of AG glycan epitopes of AGPs with antibodies

**Fig. 5:**
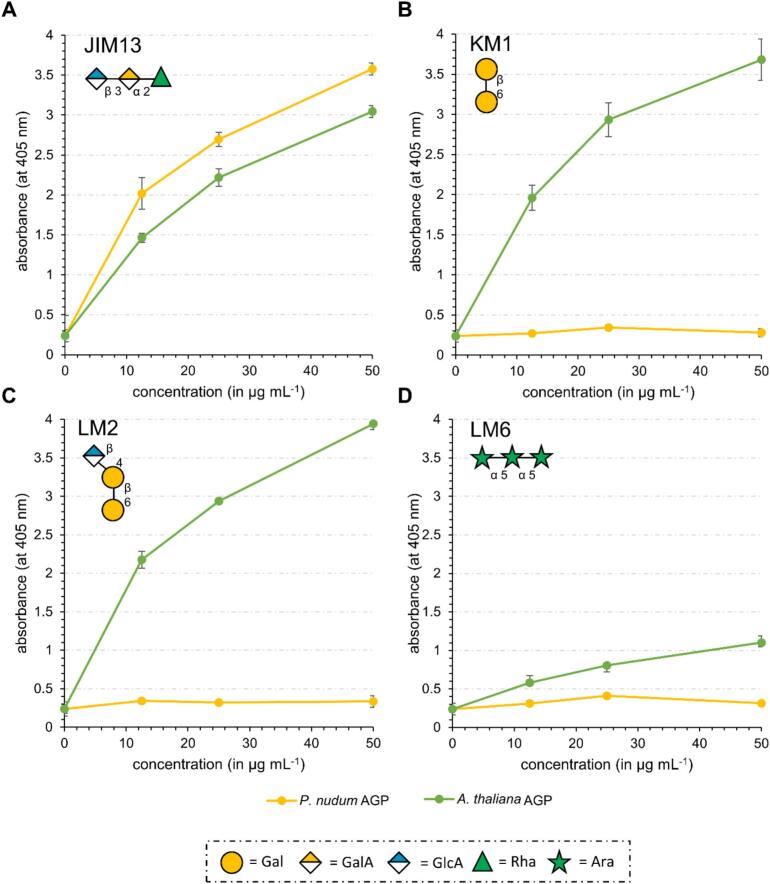
Reactivity of *Psilotum nudum* (yellow) and *Arabidopsis thaliana* (green) AGPs with antibodies against epitopes of AGP glycans (Table S3) tested by ELISA. **A**: JIM13; **B**: KM1; **C**: LM2; **D**: LM6. Error bars show the standard deviation of a three-fold measurement (error bars smaller than the dot diameter are not visible). (For interpretation of the references to colour in this figure legend, the reader is referred to the web version of this article.) Reactivity of *Psilotum nudum* (yellow) and *Arabidopsis thaliana* (green) AGPs with antibodies against epitopes of AGP glycans (Table S3) tested by ELISA. **A**: JIM13; **B**: KM1; **C**: LM2; **D**: LM6. Error bars show the standard deviation of a three-fold measurement (error bars smaller than the dot diameter are not visible). (For interpretation of the references to colour in this figure legend, the reader is referred to the web version of this article.)

Four antibodies raised against different angiosperm glycan AGP epitopes were used for detecting variations in glycan structures of the AGPs from *P. nudum* and *A. thaliana* with ELISA (Fig. 5). One of the most commonly used AGP antibodies is JIM13, although its epitope remains poorly defined. Yates et al. (1996) showed that the trisaccharide β-D-GlcpA-(1 → 3)-α-D-GalpA-(1 → 2)-α-L-Rha efficiently inhibits JIM13 binding to exudate gum antigens, and Pfeifer et al. (2022) demonstrated that Rha and GlcA contribute to antibody recognition of *Spirogyra* AGPs.

The antibody KM1 was generated against the AGP of the angiosperm *Echinacea purpurea* and detects the (1 → 6)-β-d-Gal*p* chains (Classen et al., 2004; Ruprecht et al., 2017). For LM2, an epitope consisting of (1 → 6)-β-d-Gal*p* units with terminal β-d-Glc*p*A residues was proposed (Smallwood et al., 1996; Ruprecht et al., 2017), and (1 → 5)-α-l-Ara*f* chains are crucial for the binding of the antibody LM6 (Verhertbruggen et al., 2009)*.*

As shown in Fig. 5A-D, major differences were observed in antibody binding between the two AGP preparations. All four antibodies bound to *A. thaliana* AGP, althoughthe avidity of LM6 was low. In contrast, only JIM13 reacted with *P. nudum AGP;* the other antibodies showed no reactivity, thus revealing strong differences in angiosperm and fern AGP fine structures.

### 3.4 Anatomical structure of the *P. nudum* stem with histochemical analysis and localization of AGPs

**Fig. 6:**
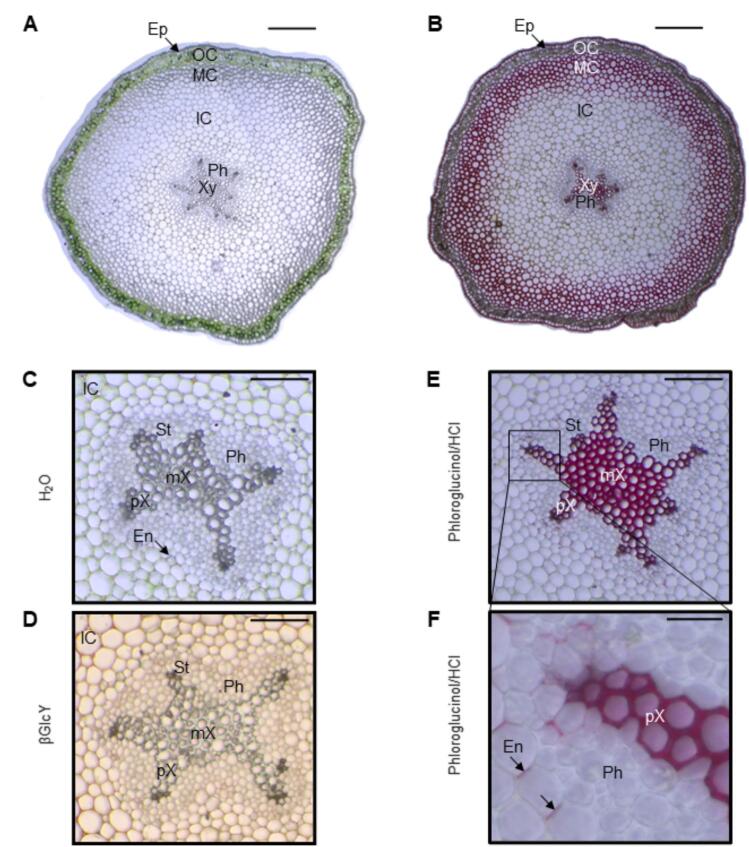
Cross sections of *Psilotum nudum* stems. **A**,**B**: Cross section in water (**A**) and after staining with phloroglucinol/HCl (**B**). **C**: Water preparation with focus on the stele with phloem and xylem surrounded by the endodermis. **D**: Stele after staining with βGlcY. **E**: Stele after staining with phloroglucinol/HCl. **F**: Enlargement of a part from **E**, arrows indicating lignin containing radial cells walls of the Casparian strip in the endodermis. Ep: epidermis; OC: outer cortex; MC: middle cortex; IC: inner cortex; En: endodermis; St: stele; pH: phloem; pX: protoxylem; mX: metaxylem; X: xylem. Scale bar: 200 μm (**A**,**B**); 50 μm (**C**-**E**); 20 μm (**F**).

**Fig. 7:**
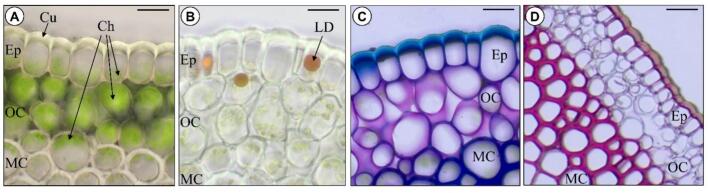
Cross sections of the periphery of *Psilotum nudum* stems, **A**: Without staining. **B**: After staining with Sudan III. **C**: After staining with toluidine blue. **D**: After staining with phloroglucinol/HCl. Ep: epidermis; OC: outer cortex; MC: middle cortex; Cu: cuticle; Ch: chloroplasts; LD: lipid droplet. Scale bar: 20 μm (**A**-**C**); 50 μm (**D**). (For interpretation of the references to colour in this figure legend, the reader is referred to the web version of this article.)

**Fig. 8:**
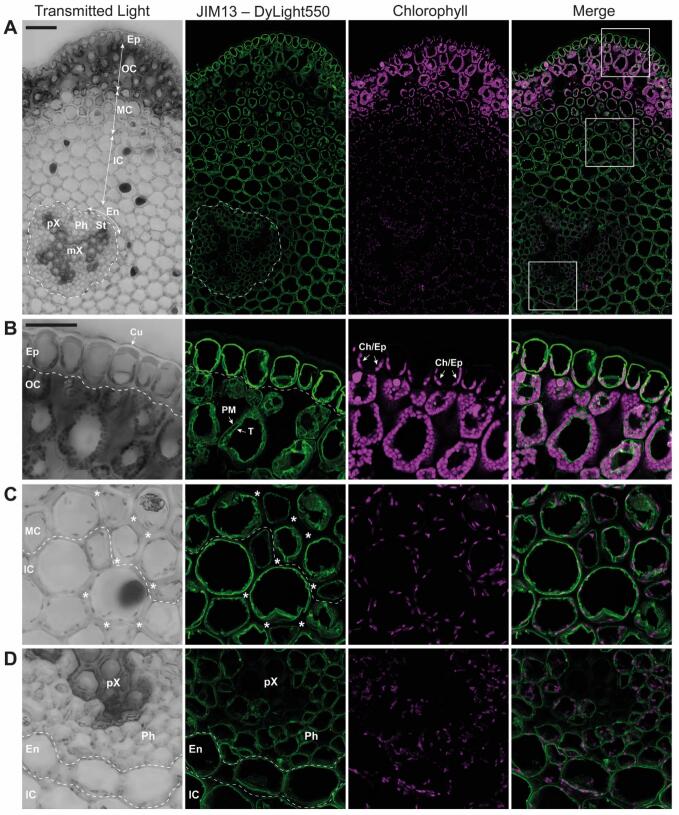
Localization of AGPs in the *Psilotum nudum* stem by JIM13 immunolabelling and confocal laser scanning microscopy. **A:** Overview of a part of a stem cross-section. Boxed areas are shown enlarged in (**B—D**). **B:** Epidermis and outer cortex (assimilating chlorenchyma). **C:** Middle cortex (sclerenchyma) and inner cortex (parenchyma). **D:** Endodermis, and phloem and xylem of the stele. Ep: epidermis; OC: outer cortex; MC: middle cortex; IC: inner cortex; En: Endodermis; St: stele; Cu: cuticle; Ch/Ep: chloroplasts in the basal part of the epidermal cells; pH: phloem; PM: plasma membrane; T: tonoplast; pX: protoxylem; mX: metaxylem. Asterisks indicate cell wall; dotted lines delineate different tissues. Scale bar: 100 μm (**A**), 50 μm (**B—D**).

Cross sections of *P. nudum* stems either unstained or after different histological stainings were examined using brightfield microscopy (Fig. 6, Fig. 7). The stem consists of the epidermis, the outer, middle and inner cortex, the endodermis and the central actinostele with xylem and phloem (Ford, 1904; Chernova et al., 2020). In epidermal cells, chloroplasts were localized to the basal part of cells (Fig. 7A and Fig. 8B, see arrows). Staining with Sudan III revealed the presence of lipid droplets in some epidermal cells (Fig. 7B). Purple staining with phloroglucinol/HCl indicated the presence of lignin in the radial and outer walls of the epidermal cells. (Fig. 7D). The outer cortex is rich in chloroplasts (Fig. 6, Fig. 7A) and seems to be mucilaginous to a certain extent, as suggested by purple staining with toluidine blue (Fig. 7C). The middle cortex consists of sclerenchyma cells, whose thickened cell walls were stained red by phloroglucinol/HCl due to their lignin content (Fig. 6B); the inner cortex is composed of parenchyma cells whose cell walls did not stain red with phloroglucinol/HCl (Fig. 6B; see also Chernova et al., 2020). The primary endodermis surrounds the star-shaped stele with phloem and lignified xylem as demonstrated by phloroglucinol/HCl staining. Cell walls of the metaxylem in the centre displayed a brighter hue than the star-shaped protoxylem arms (Fig. 6B,E,F). The Casparian strip in radial cell walls of the endodermis also stained purple with phloroglucinol/HCl, indicating the presence of lignin (Fig. 6F). Staining for AGPs with βGlcY showed slight orange coloration of the cell walls in parenchyma cells, whereas staining over the stele was not apparent (Fig. 6D).

As staining of AGPs with βGlcY is often too weak to reliably indicate the presence of AGPs, we additionally performed immunofluorescence labelling with JIM13 antibodies to localize AGPs on cross sections of the *P. nudum* stem. Results after confocal laser scanning microscopy are shown in Fig. 8; chlorophyll fluorescence indicating chloroplasts is shown in purple. Green fluorescence representing AGP that reacted with JIM13 was detected in all tissues, except some parts of the xylem (Fig. 8 A-D). While labelling was observed in the metaxylem at the centre of the stele, protoxylem was not labelled (Fig. 8A and D; Fig. S1). Regarding the subcellular localization, labelling was restricted to the area at or close to the plasma membrane. In the outer cortex, where the cytoplasmic rim is thicker due to numerous large chloroplasts, it was possible to observe that also the tonoplast was convincingly labelled (Fig. 8B). Thickened cell walls of the middle cortex were clearly not labelled (Fig. 8C). The signal over the phloem and xylem in the stele appeared weaker compared to the signal in the cortex or the epidermis (Fig. 8A).

## 4 Discussion

### 4.1 AGPs of *P. nudum* in the context of evolution

#### 4.1.1 Localization of AGPs in the *P. nudum* stem

Already in the 1970's, tests with βGlcY were established to detect AGPs in different plants, organs and tissues (Clarke et al., 1979). These tests were mostly gel diffusion assays, which are still used today (see detailed description in Happ and Classen, 2019 or Mueller et al., 2023). In these assays, a red precipitation line appears as soon as the AGP containing sample and βGlcY interact.

This interaction can also be used for detection of AGPs in plant tissue by light microscopy (Putoczki et al., 2007; Pfeifer et al., 2022). However, the staining of *P. nudum* stem cross sections with βGlcY was weak, which could mean that either βGlcY was not accessible to the cell wall to precipitate AGPs, or the content was too low to be visible by light microscopy. Possibilities to enhance sensitivity include additional use of polyclonal antibodies raised against βGlcY (Bossy et al., 2009; Goellner et al., 2013) or staining with a fluorescent azido analogue of the Yariv reagent (Rueda et al., 2023), both with the advantage that all AGPs precipitable with βGlcY are detected. Other useful tools for AGP localization are monoclonal antibodies directed against carbohydrate AGP motifs. Chernova et al. (2020) examined presence of AGPs in cross sections of *P. nudum* shoots with the monoclonal antibody JIM14, which has been raised against an AGP fraction isolated from the medium of carrot suspension culture. In cross sections of carrot root, JIM 14 labelled all tissues (Knox et al., 1991), but no labelling of the *P. nudum* stem was observed with this antibody (Chernova et al., 2020). As other antibodies raised against angiosperm AGPs did not react with *P. nudum* AGP in ELISA, it is probable that JIM14 did not bind due to differences in fine AGP structure between fern and angiosperm AGP.

As JIM13 had strong affinity to *P. nudum* AGP in ELISA, we used this antibody for fluorescence microscopy. JIM13 labelled all tissues except the protoxylem, indicating presence of AGPs in the epidermis, different tissues of the cortex, endodermis, phloem and metaxylem. Occurrence of AGPs in a huge variety of plant organs and tissues is consistent with current knowledge (Ma and Johnson, 2021). The lack of AGP labelling in *P. nudum* protoxylem is in contrast to observations in *Equisetum* stems, where AGPs were detected in cell walls of both protoxylem and metaxylem elements (Bartels, 2017). Lack of labelling of the protoxylem in *P. nudum* could be explained by the subcellular localization of the AGPs. JIM13 labelling was most intense along the plasma membrane and tonoplast, neither of which would be present in the mature protoxylem, in contrast to the slower maturing metaxylem. Therefore, we speculate that dead elements in the mature metaxylem likely no longer contain JIM13-reactive AGPs, and conversely, that JIM13-reactive AGPs may be present in the immature protoxylem elements. Also in angiosperms, AGPs have been found in xylem cells (Dolan et al., 1995; Goellner et al., 2013), and an AGP called “xylogen” mediates vascular development in *Zinnia* cell cultures (Motose et al., 2004). It is generally accepted that AGPs are cell wall glycoproteins which can be plasma membrane bound or secreted into the cell wall (Ma and Johnson, 2023). In some immunohistochemical studies, JIM13 has already been used to detect AGPs in ferns (Eeckhout et al., 2014; Lopez and Renzaglia, 2014, Lopez and Renzaglia, 2016) and seem to be important for egg and sperm cells in *Ceratopteris*, where both are covered by an AGP-rich matrix (Lopez and Renzaglia, 2014, Lopez and Renzaglia, 2016).

Furthermore, AGPs of *P. nudum* were present at the tonoplast. This has also been reported for the bryophyte *Anthoceros* (Mueller et al., 2025) and some angiosperms (Šamaj et al., 2000; Olmos et al., 2017; Sala et al., 2017). With regard to the function, Olmos et al. (2017) proposed that AGPs in tobacco cell cultures act as sodium carriers from the plasma membrane to the tonoplast during salt stress; and this unusual AGP localization in *P. nudum* proposes that there are further, still unknown functions of AGPs in ferns.

#### 4.1.2 The AGP yield of *P. nudum* in comparison to those of other ferns and *A. thaliana*

The yield of the AGPs from *P. nudum* with 0.08% of dry plant weight was lower compared to another eusporangiate fern (*Equisetum*) and four leptosporangiates (Bartels and Classen, 2017; Mueller et al., 2023) and only comparable to the semiaquatic fern *Ceratopteris* and the water fern *Salvinia* AGP (Mueller et al., 2023). The *A. thaliana* AGP yielded 0.16% and was more similar to the yields of the fern AGPs (Bartels and Classen, 2017) than to the yields of AGPs from angiosperm roots (0.01% – 0.08%, Wack et al., 2005, Thude and Classen, 2005). In general, results are difficult to compare, as AGP yield probably depend on a variety of factors like e.g. plant tissues, isolation procedures or harvest times, but an amount of water-soluble AGPs around 0.1% and 0.2% in relation to the dry plant material might be taken as a guide value.

#### 4.1.3 The protein moiety of *P. nudum* AGP: similar to those of other ferns

The protein content of the *P. nudum* (5.4%) and *A. thaliana* (7.1%) AGP was in the same range and comparable to most other fern and seed plant AGPs (5% – 12.3%; Classen et al., 2000; Bartels and Classen, 2017; Baumann et al., 2021). In contrast, the aquatic ferns *Azolla* and *Salvinia* as well as different bryophytes and the lycophyte *Lycopodium* contain a higher protein content (15% – 25%; Bartels et al., 2017; Bartels and Classen, 2017; Happ and Classen, 2019; Mueller et al., 2023, Mueller et al., 2025).

The Hyp content of the *A. thaliana* AGP protein moiety (8.7%) corresponded to those of other dicotyl angiosperms (between 6.3% and 17.3%; Wack et al., 2005; Thude and Classen, 2005; Bossy et al., 2009). However, the Hyp amount of the whole protein of the eusporangiate *P. nudum* AGP (6.1%) is more similar to those of the leptosporangiate AGPs (1.5% – 6.1%) than to the high proportion found in *Equisetum* AGP (15.9%; Bartels and Classen, 2017; Mueller et al., 2023).

#### 4.1.4 The carbohydrate moiety of *P. nudum* AGPs: a mixture of features found in leptosporangiate fern and lycophyte AGPs

The structure of *A. thaliana* leaf AGP has been investigated in-depth before (Tryfona et al., 2012) and showed the typical galactan core structure known from seed plants (Seifert and Roberts, 2007). Here, the results for the arabinogalactan moiety of the *A. thaliana* AGP from aerial parts were in good correspondence with findings on *A. thaliana* leaf AGP (Tryfona et al., 2012) with the exception that we detected only traces of terminal Fuc*p*. In *P. nudum* AGP, the 1,3-Gal*p* linkages were slightly more abundant and the 1,6-Gal*p* lower compared to dicot AGPs (Classen et al., 2000; Thude and Classen, 2005; Wack et al., 2005; Mueller et al., 2023). This low content was confirmed by the negative affinity of the KM1 antibody via ELISA, which detects (1 → 6)-β-d-Gal*p* chains (Classen et al., 2004; Ruprecht et al., 2017). We hypothesize that low amounts of Gal*p* in 1,6-linkage are a typical feature for spore plant AGPs, as low amounts have been already identified in seven fern (1.3% – 2.9%; Bartels and Classen, 2017; Mueller et al., 2023), two lycophyte (0% – 1.1%; Bartels and Classen, 2017, Schuldt, 2021) and five bryophyte AGPs (trace-2.6%; Bartels et al., 2017; Happ &Classen, 2019; Mueller et al., 2025). In contrast, a high portion of 1,6-linked Gal has been found in *A. thaliana* AGP (14.7% in this study; high amounts also in *A. thaliana* leaf AGP; see Tryfona et al., 2012) comparable to *Echinacea* AGP (15.4%; Classen et al., 2000).

In *P. nudum* and *A. thaliana* AGPs, 1,6-Gal*p* as well as the terminal 1-Gal*p* linkages increased after partial oxalic acid hydrolysis. It can be concluded that labile Ara residues, removed by this reaction, were attached to C3 and/or C6 of the 1,3,6-Gal*p* linkages in the native AGPs.

The galactan core structure of seed plants is mainly decorated with terminal Ara*f* (Ma et al., 2018) and sometimes longer chains of Ara. The linkage of these chains differs within the land plant AGPs. In angiosperm AGPs, 1,5-Ara*f* seems to be most abundant (Goellner et al., 2010; Mueller et al., 2023; also proposed by Tryfona et al., 2012), and this has been confirmed also in this study for *A. thaliana* AGP by methylation analysis and ELISA with the antibody LM6 (Verhertbruggen et al., 2009).

In *P. nudum* AGP, we detected mainly 1,3-Ara*f*, which is in contrast to *Equisetum* AGP (mainly 1,5-Ara*f*) and to leptosporangiate fern AGPs (mainly 1,2-Ara*f*; Mueller et al., 2023), but in correspondence with results on two lycophyte AGPs, which also contained high amounts of 1,3-linked Ara*f* (Bartels and Classen, 2017; Schuldt, 2021). Terminal Ara residues in AGPs are generally in furanosidic ring form (Ma and Johnson, 2023). Therefore, it is an unusual feature that *P. nudum* AGP contained more terminal Ara*p* than terminal Ara*f*, especially as we found the same for two lycophyte AGPs from *Lycopodium* and *Huperzia* (Bartels and Classen, 2017; Schuldt, 2021).

A further difference of the *P. nudum* AGP to the AGPs of the most bryophytes and ferns is the lack of terminal 3-*O*-MeRha*p* (Akiyama et al., 1987; Popper et al., 2004; Fu et al., 2007; Bartels and Classen, 2017; Bartels et al., 2017; Happ and Classen, 2019; Ali et al., 2025). *Lycopodium* and *Huperzia* AGPs are characterized by complete lack of Rha (Bartels and Classen, 2017; Schuldt, 2021). However, in the *P. nudum* AGP, terminal unmethylated Rha*p* was found. High affinity of JIM13 to the *P. nudum* AGP confirmed presence of Rha residues. As mentioned above, this antibody likely recognizes epitopes including Rha and GlcA (Pfeifer et al., 2022). GlcA was identified as t-Glc*p*A and 1,4-Glc*p*A residues in *P. nudum* AGP and as t-Glc*p*A and t-4-*O*-MeGlc*p*A in that of *A. thaliana*. These terminal residues were also identified in *A. thaliana* leaf AGPs (Tryfona et al., 2012).

**Fig. 9:**
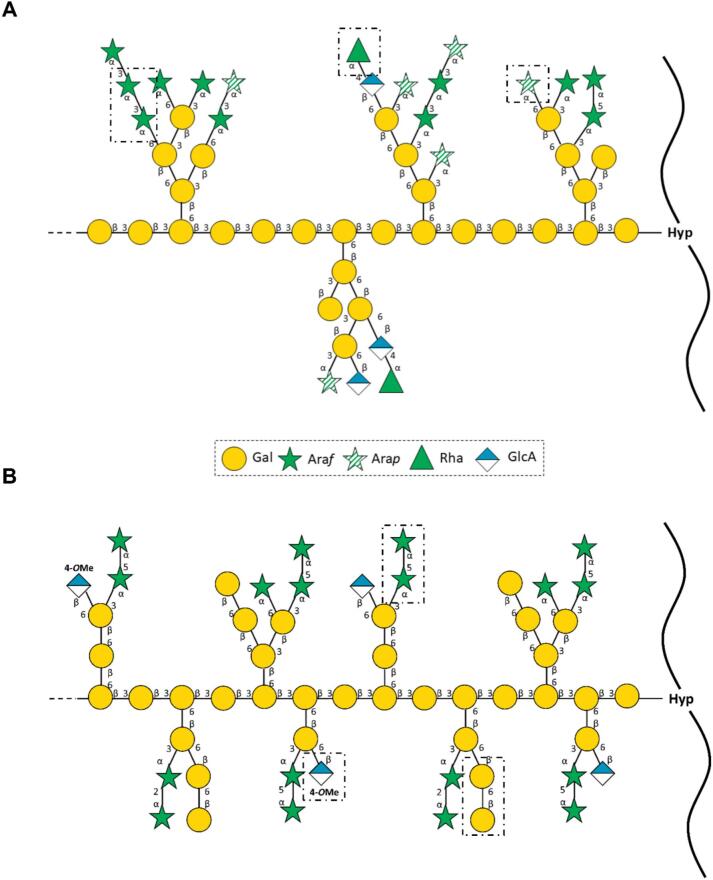
Structural proposal of the carbohydrate moiety of the AGP from *Psilotum nudum* (**A**) and *Arabidopsis thaliana* (**B**), based on compositional and linkage-type analyses of the native AGP, the reduced AGP_UR_, and the hydrolysed AGP_UROx_. Most significant structural differences are shown in dashed lines and comprise for *P. nudum* AGP: 1,3-Ara*f*, t-Rha*p*, t-Ara*p*; for *A. thaliana* AGP: 1,5-Ara*f*, 1,6-Gal*p* and t-4-*O*-MeGlc*p*A.

Based on these findings, a structural proposal for *P. nudum* in comparison to *A. thaliana* AGP is shown in Fig. 9, which reveals the most significant structural differences:

- *P. nudum* AGP with 1,3-linked Ara*f,* terminal Rha*p* and terminal Ara*p*
- *A. thaliana* AGP with 1,6-linked Gal*p*, 1,5-linked Ara*f* and terminal 4-*O*-MeGlc*p*A.

**Fig. 10:**
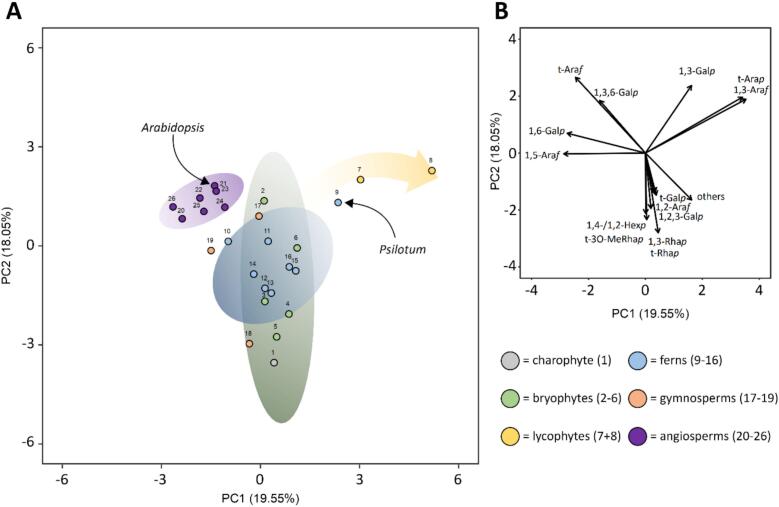
Principal component analysis (PCA) of different AGPs throughout the streptophyte lineage. (A) Two-dimensional projection of the sample positions based on linkage-type analyses. For three groups (angiosperms, ferns and bryophytes), shaded areas based on the 0.90 confidence interval are shown. The lycophyte position is highlighted with a yellow shaded arrow. (B) Loading vectors of the calculated PCA. The numbers shown in brackets after the lineage name refer to the sample number shown in panel (A) as well as in Table S4. (For interpretation of the references to colour in this figure legend, the reader is referred to the web version of this article.) Principal component analysis (PCA) of different AGPs throughout the streptophyte lineage. (A) Two-dimensional projection of the sample positions based on linkage-type analyses. For three groups (angiosperms, ferns and bryophytes), shaded areas based on the 0.90 confidence interval are shown. The lycophyte position is highlighted with a yellow shaded arrow. (B) Loading vectors of the calculated PCA. The numbers shown in brackets after the lineage name refer to the sample number shown in panel (A) as well as in Table S4. (For interpretation of the references to colour in this figure legend, the reader is referred to the web version of this article.)

The implementations with regard to evolution of AGPs in land plants were demonstrated in a principal component analysis of AGP fine structures (Fig. 10).

In a recent paper (Mueller et al., 2023), we compared 21 species of the streptophyte lineage regarding their AGP carbohydrate fine structure. We now added five further species: the hornwort *Anthoceros agrestis*, the moss *Physcomitrium patens*, the lycophyte *Huperzia squarrosa*, the eusporangiate fern *Psilotum nudum* and the angiosperm *Arabidopsis thaliana*. Structure of angiosperm AGPs from monocots to dicots seems to be quite conserved, whereas bryophyte, lycophyte and fern AGPs show higher diversity of AGP structure. The position of *P. nudum* AGP is closer to the lycophytes as to seed plants, although ferns are more closely related to seed plants than to lycophytes (PPG, 2016). Furthermore, *P. nudum* AGP structure is closer to leptosporangiate ferns as to the other eusporangiate fern, *Equisetum.* In fact, the phylogenetic position of the Equisetales is still under discussion, e.g. as the sister to Psilotales + Ophioglossales (Nitta et al., 2022) or as the sister group to the last common ancestor of all remaining ferns (Ali et al., 2025). Therefore, this study's characterization of AGPs from the cell wall of *P. nudum* provides new insights into the evolution of land plant AGPs and raises further questions regarding the phylogenetic position of *P. nudum*.

The glycan parts of bryophyte, lycophyte and fern AGPs investigated up to now are decorated with high amounts of 3-*O*-MeRha*p*, Rha*p* or Ara*p*. These terminal residues are more hydrophobic in comparison to Ara*f* and Glc*p*A, which dominate in seed plant AGPs. This shift to a more hydrophilic glycan surface seems to be a result of adaptation to land through evolutionary pressure. For other macromolecular structures, like e.g. LEA proteins, a similar shift towards greater hydrophilicity was observed during land plant evolution (Battaglia et al., 2008). The achieved greater hydrophilicity acts there as a protective mechanism against abiotic factors like cold, drought or high salinity (Chen et al., 2019). AGP surface modifications through 4-*O*-methylation of Glc*p*A residues are described (Temple et al., 2019) and act there as important influential factors for AGP‑calcium interaction (Lopez-Hernandez et al., 2020) or pollen-tube guidance (Mizukami et al., 2016). In general, in algal polysaccharides methylated carbohydrates are more frequently (Staudacher, 2012). This modification (often methylated galactose residues) is described to stabilize the polysaccharides by providing resistance against microbial degradation (Reisky et al., 2018). Further AGPs from algae and plants of the streptophyte lineage have to be analytically and functionally characterized to answer the question, why structural changes of AGPs were necessary during evolution and what are the implications with regard to functions of these fascinating complex macromolecules.

## CRediT authorship contribution statement

**Kim-Kristine Mueller:** Writing – review & editing, Writing – original draft, Visualization, Methodology, Investigation, Formal analysis, Data curation, Conceptualization. **Lukas Pfeifer:** Writing – review & editing, Visualization, Methodology, Investigation, Formal analysis, Data curation. **Urska Repnik:** Writing – review & editing, Visualization, Investigation, Data curation. **Marc Bramkamp:** Writing – review & editing, Visualization, Investigation, Data curation. **Birgit Classen:** Writing – review & editing, Writing – original draft, Supervision, Resources, Project administration, Methodology, Funding acquisition, Formal analysis, Conceptualization.

## Declaration of competing interest

The authors declare that they have no known competing financial interests or personal relationships that could have appeared to influence the work reported in this paper.

## Data Availability

Data will be made available on request.
