# Supplementary material for: Arabinogalactan-proteins of the eusporangiate fern Psilotum nudum show atypical structural features compared to other ferns
Source: Cell Surf. 2025 Oct 16;14:100157. doi: 10.1016/j.tcsw.2025.100157 (PMC12590268; doi:10.1016/j.tcsw.2025.100157)
Supplement: Supplementary Tables S1-4 and Supplementary Figure S1 [file mmc1.pdf]

Supplementary data to:

Arabinogalactan-proteins of the eusporangiate fern *Psilotum nudum* show atypical structural features compared to other ferns

Table S1. Neutral monosaccharide composition of AE, AGP, AGP_{Ur} and AGP_{UrOx} of *Psilotum nudum* in % (mol mol^{-1}; n=3).

Neutral monosaccharide	AE	AGP	AGP_{Ur}	AGP_{UrOx}
Rha	4.0 ± 0.1	4.7 ± 0.1	4.5 ± 0.2	3.3 ± 0.2
Fuc	1.2 ± 0.1	tr	-	-
Rib	tr	-	-	-
Ara	26.7 ± 0.8	36.6 ± 0.8	36.0 ± 0.6	11.2 ± 0.1
Xyl	3.2 ± 0.2	-	-	-
Man	10.6 ± 1.0	tr	tr	-
Gal	34.9 ± 1.6	54.7 ± 0.5	54.6 ± 0.3	78.8 ± 0.4
4-*O*-MeGlc	-	-	-	-
Glc	19.4 ± 1.1	4.0 ± 0.3	4.9 ± 0.3	6.7 ± 0.3
Ara : Gal	1 : 1.3 ± 0.0	1 : 1.5 ± 0.0	1 : 1.5 ± 0.0	1 : 7.0 ± 0.1

tr: trace value < 1 %

Table S2. Neutral monosaccharide composition of AE, AGP, AGP_{Ur} and AGP_{UrOx} of *Arabidopsis thaliana* in % (mol mol^{-1}; n=3).

Neutral monosaccharide	AE	AGP	AGP_{Ur}	AGP_{UrOx}
Rha	7.4 ± 0.7	tr	1.0 ± 0.0	tr
Fuc	tr	tr	tr	tr
Rib	tr	-	-	-
Ara	21.3 ± 0.6	36.6 ± 1.1	34.6 ± 0.3	10.7 ± 0.3
Xyl	11.1 ± 1.0	tr	tr	-
Man	5.8 ± 4.8	tr	tr	tr
Gal	24.5 ± 2.4	62.3 ± 1.1	60.0 ± 0.2	86.5 ± 0.1
4-*O*-MeGlc	-	-	2.0 ± 0.3	-
Glc	29.9 ± 1.4	1.1 ± 0.1	2.4 ± 0.1	2.8 ± 0.1
Ara : Gal	1 : 1.2 ± 0.1	1 : 1.7 ± 0.1	1 : 1.7 ± 0.0	1 : 8.1 ± 0.2

tr: trace value < 1 %

Table S3. Antibodies tested for binding to glycan epitopes of *Psilotum nudum* and *Arabidopsis thaliana* AGP.

Antibody	Epitope	Key References
JIM13	AGP glycan,<br>e.g. β-D-Glc*p*A-(1→3)-α-D-Gal*p*A-(1→2)-α-L-Rha	Yates *et al*. (1996); Pfeifer *et al*. (2022)
KM1	(1→6)-β-D-Gal*p* units in AGs type II	Classen *et al*. (2004); Ruprecht *et al*. (2017)
LM2	(1→6)-β-D-Gal*p* units with terminal ß-D-Glc*p*A in AGP	Smallwood *et al*. (1996); Ruprecht *et al*. (2017)
LM6	(1→5)-α-L-Ara*f* oligomers in arabinan or AGP	Verhertbruggen *et al*. (2009)

Table S4. Linkage type analysis of AGPs throughout the streptophyte lineage in % (mol mol^{-1}).

		1,3,6-Gal*p*	1,2,3-Gal*p*	1,3-Gal*p*	1,6-Gal*p*	t-Gal*p*	1,2-Ara*f*	1,3-Ara*f*	1,5-Ara*f*	t-Ara*f*	t-Ara*p*	t-Rha*p*	t-3-O-MeRha*p*	1,3-Rha*p*	1,4-/1,2-Hex*p*	others
Charophyte alga	*S. pratensis*	30.7	0	9.8	5.8	0	0	0	0	1.1	0	24.7	2.0	20.0	0	5.9
Bryophytes	*A. agrestis*	24.0	0	18.0	1.0	1.0	0	2.0	0.5	26.0	2.0	0.5	6.5	0	0	18.5
	M. polymorpha	27.1	0	19.2	0	0	0	4.3	2.7	36.3	0	1.1	1.1	0	4.3	3.9
	P. patens	18.0	0	11.0	1.0	2.0	0	0.5	13.0	10.0	0.5	4.8	13.2	0	0	26.0
	P. formosum	28.2	6.6	14.5	2.6	14.2	0.6	3.4	1.8	9.2	0	7.4	2.5	0	6.8	2.2
	Sphagnum sp.	27.5	4.8	17.8	7.3	7.0	0	0.5	5.3	3.9	0	1.9	7.2	0	11.1	5.7
Lycophytes	*L. annotinum*	29.2	0	19.5	1.1	1.3	0.2	15.2	1.0	11.8	13.4	0	0	0	3.1	4.2
	H. squarrosa	22.7	0	18.7	0	0	0	24.4	0	5.0	20.9	0	0	0	0.5	7.8
Ferns	*P. nudum*	33.5	0	20.5	2.9	4.2	0	10.8	1.1	7.1	9.5	3.4	0	0	0	7.0
	E. arvense	26.4	0	16.3	1.8	2.4	0.5	0.2	14.3	23.1	0	0	3.4	0	5.2	6.4
	O. regalis	35.5	0	19.7	1.3	3.4	2.9	0.8	2.4	15.9	0	1.1	5.1	0	6.3	5.6
	S. molesta	30.7	0	15.2	2.1	2.1	17.2	0	3.9	9.6	0	5.4	5.6	0	5.3	2.9
	A. filiculoides	27.0	0	15.5	2.6	3.4	16.9	0	4.4	8.5	0	9.5	0	0	7.9	4.3
	C. richardii	35.9	0	16.4	2.2	6.0	11.5	0	7.3	6.4	0	0	7.8	0	3.0	3.5
	P. aquilinum	29.7	0	20.6	2.2	10.6	9.6	1.7	2.5	4.6	0	0.5	1.3	0	7.6	9.1
	D. filix-mas	23.7	0	18.5	2.9	9.0	5.0	1.7	2.9	15.2	0	0.7	1.3	0	4.8	14.3
Gymnosperms	*C. revoluta*	32.4	0	13.2	3.8	2.1	1.5	7.1	6.9	21.7	3.7	4.4	0	0	1.1	2.1
	G. biloba	12.6	0	2.8	1.3	2.3	1.0	2.3	13	8.4	1.0	2.1	1.2	0	47.3	4.7
	E. distachya	39.2	0	7.3	4.0	0.9	0.7	0	3.7	27.4	0.5	2.4	8.7	0	5.2	0
	A. thaliana	39.4	0	10.9	14.7	3.8	1.2	0.5	9.0	17.0	0	0.5	0	0	0	3.0
	T. aestivum	37.7	0	19.0	3.0	1.2	0	0	9.0	30.1	0	0	0	0	0	0
	S. cereale	36.3	0	14.2	6.4	3.3	0	0	7.7	32.1	0	0	0	0	0	0

Angiosperms	*A. sativa*	36.8	0	19.6	6.1	3.6	0	0	7.3	26.6	0	0	0	0	0	0
	B. tinctoria	24.2	0	16.7	5.8	3.6	0	1.7	9.4	33.2	1.9	1.7	0	0	1.8	0
	E. pallida	26.9	0	16.5	6.3	2.5	0	0	14.2	29.4	0	0	0	0	0	4.2
	E. purpurea	31.8	0	12.7	15.4	0	0	0	11.7	24.8	0	0	0	0	0	3.6

References: Schuldt, 2021; Mueller *et al*., 2023, 2025

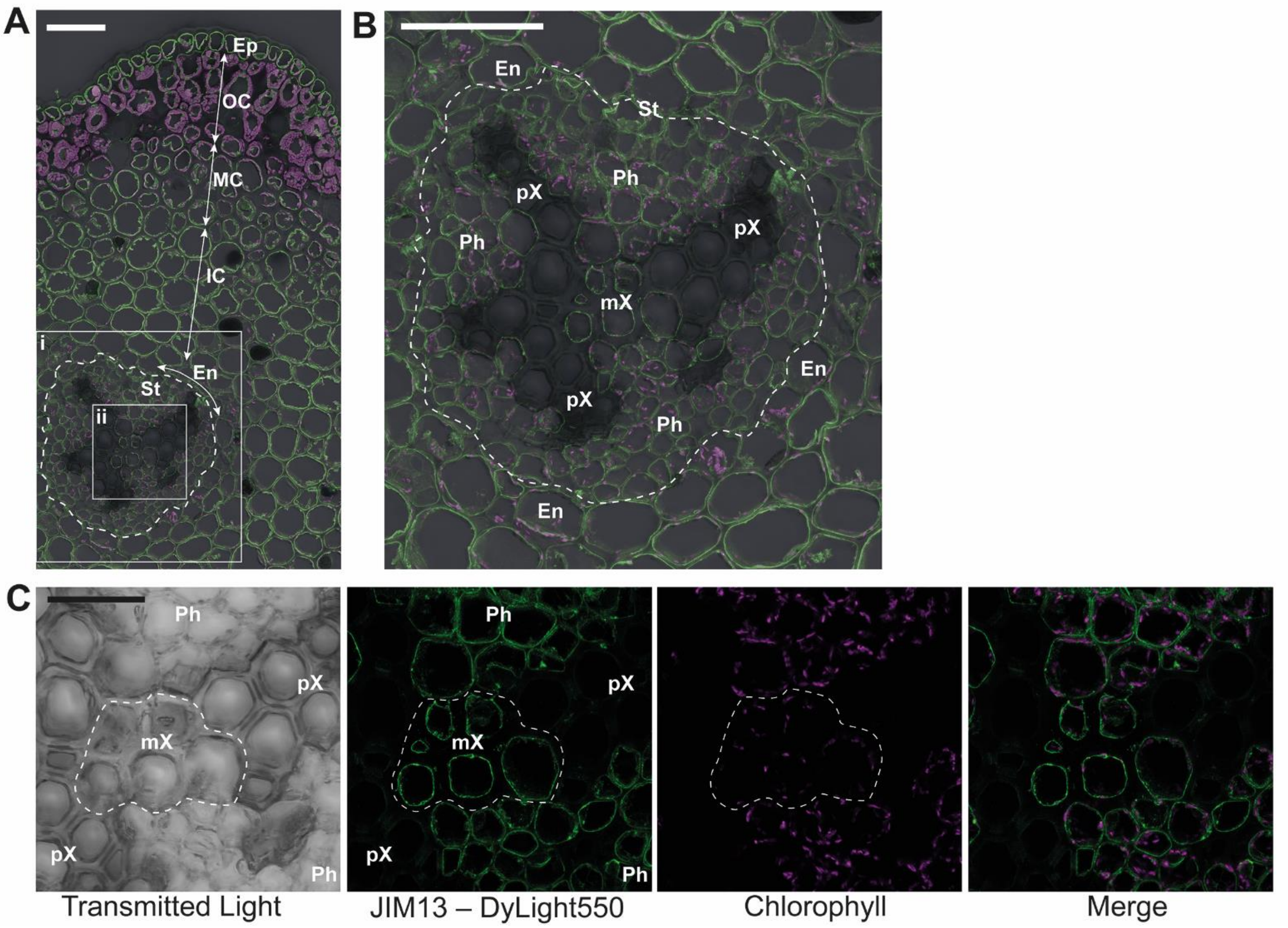

Fig. S1. Localization of AGPs in the *Psilotum nudum* stem by JIM13 immunolabelling and confocal laser scanning microscopy. (A,B) Overlay of the transmitted light and fluorescence images. (C) Transmitted light image, individual fluorescence images for distinct signals, and a merged fluorescence image. Box (i) is shown enlarged in (B), box (ii) is shown enlarged in (C). The arms representing the primary protoxylem are not labelled with JIM13, whereas the metaxylem in the centre of the stele is labelled. Labelled xylem elements are alive as indicated by the presence of chloroplasts. Ep: epidermis; OC: outer cortex; MC: middle cortex; IC: inner cortex; En: Endodermis; St: stele; Ph: phloem; pX: protoxylem; mX: metaxylem. Dotted lines delineate different tissues. Scale bar: 100 µm (A,B), 50 µm (C).
